# Evaluation of Periodontal Clinicoradiographic Status and Whole Salivary Prostaglandin E2 Levels among Users of Water Pipe and Cigarettes

**DOI:** 10.3290/j.ohpd.b5629079

**Published:** 2024-07-26

**Authors:** Asmaa Saleh Almeslet, Suha Mohammed Aljudaibi, Mohammad Abdullah Zayed Alqhtani, Abdulrahman Ahmed Aseri, Sultan Mohammed Alanazi

**Affiliations:** a Associate Professor, Department of Oral Maxillofacial Surgery and Diagnostic Sciences, College of Medicine and Dentistry, Riyadh Elm University, Riyadh, Saudi Arabia. Wrote the manuscript, revised the draft before submission.; b Assistant Professor, Department of Preventive Dental Sciences, College of Dentistry, Princess Nourah bint Abdulrahman University, Riyadh, Saudi Arabia. Wrote the manuscript, revised the draft before submission, funding acquisition.; c Assistant Professor, Prosthetic Dental Science Department, Faculty of Dentistry, Najran University, Najran, Saudi Arabia. Wrote the manuscript, revised the draft before submission.; d Assistant Professor, Department of Preventive Dental Sciences, Faculty of Dentistry, Najran University, Najran, Saudi Arabia. Wrote the manuscript, revised the draft before submission.; e Assistant Professor, Department of Preventive Dental Sciences, Faculty of Dentistry, Najran University, Najran, Saudi Arabia. Wrote the manuscript, revised the draft before submission.

**Keywords:** alveolar bone loss, cigarette smoking, periodontal inflammation, prostaglandin E2, unstimulated whole saliva, water pipe

## Abstract

**Purpose:**

The objective was to evaluate the periodontal clinicoradiographic status and whole salivary prostaglandin E2 (PgE2) levels among users of water pipe and cigarettes.

**Materials and Methods:**

Demographic data, duration of smoking (pack years), and familial history of smoking were recorded using a questionnaire. Participants were allocated into three groups based on their smoking status: group 1: self-reported cigarette smokers (CS); group 2: self-reported water-pipe-users; and group 3: non-smokers. The assessment included measurements of full-mouth plaque and gingival indices (PI and GI), as well as probing depth (PD), clinical attachment loss (CAL), and marginal bone loss (MBL). Unstimulated whole saliva samples were collected and PgE2 levels were measured. Group comparisons were done and p<0.05 was considered statistically significant.

**Results:**

Thirty-three, 34 and 33 individuals were included in groups 1, 2 and 3, respectively. Full mouth PI (p<0.05), GI (p<0.05), PD (p<0.05) and mesial (p<0.05) and distal (p<0.05) MBL were statistically significantly higher among patients in groups 1 and 2 than group 3. The scores of CAL in groups 1 and 2 were 3.45 ± 0.97 and 3.62 ± 1.2 mm, respectively. None of the individuals in the control group displayed CAL. PgE2 levels were statistically significantly higher among patients in groups 1 (231.5 ± 66.3 pg/ml) (p<0.05) and 2 (231.5 ± 66.3 pg/ml) (p<0.05) compared with group 3 (76.6 ± 10.6 pg/ml). In groups 1 and 2, a statistically significant relationship was observed between pack-years, the duration of water-pipe smoking, and the levels of PgE2 and PD.

**Conclusion:**

There is no difference in periodontal clinicoradiographic status and whole salivary PgE2 levels between CS and waterpipe-users; however, these parameters are worse in CS and water-pipe users than in non-smokers.

Gingivitis (clinically manifested as gingival redness and bleeding gums) and a probing depth (PD) of 3 mm or greater are typical clinical manifestations of periodontal inflammation, a prevalent condition in the field of oral health.^35^ A suboptimal state of oral hygiene is a common, localised risk factor for the development of periodontal inflammation;^20^ however, habits such as cigarette smoking have also been linked with the aetiopathogenesis of periodontal diseases.^11,26^ Cigarette smokers (CS) are usually well aware of the detrimental effects of smoking on oral and overall health;^4,5^ however, quitting the habit is often challenging due to its withdrawal symptoms such a nervousness, constipation and anxiety.^1,8^ Individuals trying to reduce or quit their cigarette smoking habit often turn to alternative methods of nicotine consumption, and one such approach is the use of a water pipe, also referred to as a hubble-bubble, hookah, shisha, or narghile.^7,33,36^ Studies^4,5,32,33^ have shown that scores of periodontal inflammatory parameters, including plaque index (PI), gingival index (GI), PD and clinical attachment loss (CAL), are elevated in CS and water-pipe users compared with non-smokers. Moreover, scientific evidence supports the notion that water-pipe usage is not less detrimental to general and periodontal health than cigarette smoking.^4,5,32,33^

Prostaglandin E2 (PgE2) is a biologically active lipid that plays a central role in a diverse array of physiological and pathological processes.^15,39,43^ The distinct functions of PgE2 encompass regulation of inflammation and immune response, vasodilation, and pain perception.^15,27,38^ In periodontal research, a limited number of studies^18,19,40^ have correlated periodontal inflammation with the expression of PgE2 in unstimulated whole saliva (UWS). Sánchez et al^40^ reported that whole salivary PgE2 levels are stastistically significantly higher in patients with than without periodontitis. Likewise, Gümüş et al^18^ compared whole salivary PgE2 levels in patients with chronic periodontitis and individuals with a healthy periodontal status,^18^ finding stastistically significantly higher whole salivary PgE2 levels in patients with periodontitis in contrast to individuals a healthy periodontal status. Moreover, in that study,^18^ a correlation existed between whole salivary PgE2 levels and PI. As CS and water-pipe use are widely recognised as risk factors for periodontal inflammation,^21,23^ and previous studies^5,12,25,33,37^ have indicated that periodontal inflammation is more severe in CS and water-pipe users compared to controls, it has been hypothesised that salivary PgE2 levels are elevated in CS and water-pipe users compared to controls. However, a thorough examination of the relevant literature has revealed a gap in research, as there are currently no studies that have investigated and compared salivary PgE2 levels in unstimulated whole saliva samples from cigarette smokers and water-pipe users.

The aim of the present study was therefore to evaluate the periodontal, clinicoradiographic status and whole salivary PgE2 levels among CS, water-pipe users and non-smokers.

## MATERIALS AND METHODS

### Ethical Approval

The ethics committee at Riyadh Elm University, Riyadh, Saudi Arabia reviewed and approved the present study (FRP/2023/522). Participation was optional, and all individuals had the option to discontinue their involvement at any stage during the research. Participants’ data such as name, address, and contact details were kept confidential. All volunteering participants were encouraged to pose any questions or inquiries they had. Those who chose to volunteer were asked to carefully review and sign a written informed consent.

### Study Location and Design

The study was performed at the Department of Oral and Maxillofacial Surgery and Diagnostic Sciences, Riyadh Elm University, Riyadh, Saudi Arabia. The present investigation had a cross-sectional comparative study in which self-reported CS, water-pipe users and individuals who reported to have never used any form of nicotinic products – combustible or/and non-combustible – (controls) were included.

### Inclusion and Exclusion Criteria

The eligibility criteria comprised: (a) adults aged 18 years or older; (b) self-reported cigarette smokers (those reporting daily smoking of at least one cigarette over the past 12 months);^24^ (c) self-reported water-pipe users (those using water pipe at least once daily over the past 12 months);^3^ (d) self-reported non-smokers/controls.^24^ Exclusion criteria encompassed: (a) individuals with self-reported systemic diseases such as hepatic/renal disorders, diabetes mellitus (DM), viral infections, cardiovascular diseases, respiratory diseases, arthritis and HIV/AIDS. Additionally, pregnant and/or nursing females and habitual alcohol users were not included. From a dental perspective, individuals who were completely edentulous in both arches, or individuals with third molars, supernumerary teeth, or severely decayed teeth with embedded root remnants were excluded and categorised as “missing teeth.”

### Questionnaire

The principal investigator administered a questionnaire to all participants, collecting relevant information on duration of cigarette smoking in pack years (PY), age, gender, duration and daily frequency of water-pipe usage (number of times daily), duration of each water-pipe session in minutes, and any familial history of smoking. Participants were also asked about their daily frequencies of toothbrushing and flossing. Cigarette smokers were subdivided into three subgroups: light smokers (up to 20 PY), moderate smokers (20.1-40.0 PY), and heavy smokers (more than 40 PY).^29^

### Study Groups

Participants were divided into three groups based on their smoking status: group 1: self-reported cigarette smokers; group 2: self-reported water-pipe users; and group 3: non-smokers (controls).

### Clinical and Radiographic Periodontal Examination

In all patients, the assessment of clinical periodontal parameters included: PI,^31^ GI,^41^ PD,^10^ and CAL.^13^ The PI and GI were measured at four sites per tooth (mesial, distal, lingual/palatal, and facial/buccal). PD was evaluated at six sites per tooth (mesiobuccal, midbuccal, distobuccal, mesio lingual/palatal, midlingual/palatal, and distolingual/palatal) using a sterile graded probe (UNC 15; Chicago, IL, USA). Marginal bone loss (MBL) was assessed on the mesial and distal surfaces of all teeth through digital intraoral radiographs, taken using the long cone paralleling technique. MBL was recorded as the linear distance from 2 mm below the cementoenamel junction to the alveolar crest.^24^ All clinical and radiographic evaluations were performed by a single blinded, trained, and calibrated investigator, with Kappa scores of 0.88 and 0.9 for clinical and radiographic assessments, respectively.

### Collection of Unstimulated Whole Saliva Samples and Assessment of Prostaglandin E2 Levels

The UWS samples were obtained during early morning hours with patients in a fasting state. An established protocol as outlined in previous literature was adopted.^6,22^ In a quiet setting, participants were seated and asked to refrain from swallowing and jaw movements for 5 minutes. The patients were then requested to expectorate the saliva accumulated in the mouth during this period into a graduated cylinder through a disposable plastic funnel. The unstimulated whole salivary flow rate was promptly calculated. The collected UWS samples were immediately transferred to sterile plastic tubes with lids and centrifuged at 2000xg for 15 min in a cold room (4°C). The resulting supernatant was stored at -70°C, with all UWS supernatants analysed for PgE2 levels within 24 h of collection. The UWS and corresponding supernatant samples were obtained by a blinded investigator, with Kappa scores of 0.88 and 0.86, respectively. Whole salivary PgE2 levels were determined following the methodology described in a previous study.^19^ In brief, duplicate wells were used to analyse whole salivary PgE2 levels with a human PgE2 enzyme-linked immunosorbent assay (ELISA) kit (Biosource, Invitrogen; Carlsbad, CA, USA), following the manufacturer’s instructions. Laboratory-based investigations were conducted by a calibrated investigator who remained blinded to the study groups, achieving a Kappa score of 0.92.

### Sample-size Estimation and Statistical Analyses

Data derived from a preliminary pilot investigation was used to estimate the sample size using nQuery Advisor 6.0 (Statistical Solutions; Saugas, MA, USA). The targeted level of statistical power was set at 0.90, indicating a 90% probability of detecting a true effect if present, with an alpha level of 5% designating the threshold for statistical significance. To discern a difference of 2 mm in PD among the study groups, it was determined that a minimum of 30 patients should be included in each group. All statistical analyses were executed using SPSS software, version 22 (Chicago, IL, USA). The Shapiro-Wilk test was employed to assess the normality of the data. Means and standard deviations for clinical, radiographic, and laboratory-based parameters were calculated, and group comparisons were conducted using one-way ANOVA with Bonferroni post-hoc adjustment. Logistic regression models were utilised to evaluate correlations between clinico-radiographic parameters and whole salivary PgE2 levels. Statistical significance was established at p<0.05. Intra-examiner reliability was gauged through repeated measurements on a subset of patients, and the kappa score was calculated to assess the consistency of measurements.

## RESULTS

### Study Cohort

**Fig 1:**
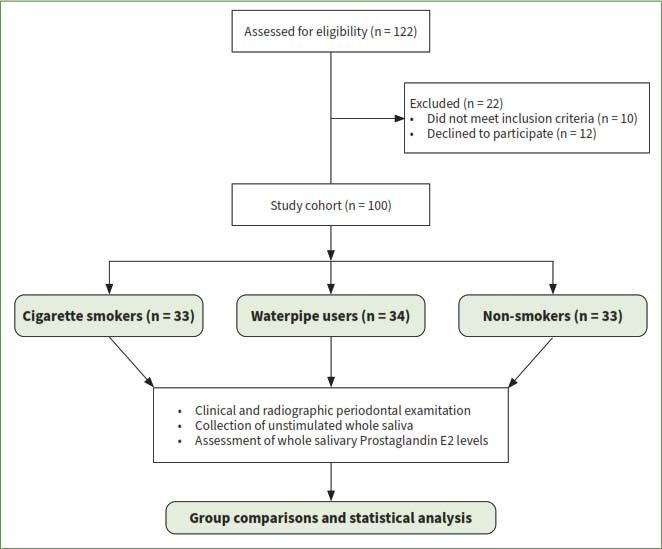
Consort flow diagram.

An initial cohort comprising 122 individuals was extended invitations to participate. Twenty-two (22) individuals who failed to meet the predetermined eligibility criteria were subsequently excluded. Consequently, the study encompassed a final sample size of 100 individuals, stratified into the following three groups: group 1: n=33 CS; group 2: n=34 water-pipe users; and group 3: n=33 non-smokers, controls (Fig 1). There was no statistically significant difference in the mean age of individuals in all groups. In groups 1, 2 and 3, 25, 30 and 30 participants, respectively, were males. In group 1, participants had a cigarette-smoking history of 9.4 ± 3.9 PY and were therefore categorised as mild CS. In group 2, participants reported to have been using water pipe for an average duration of 13.5 ± 3.3 years. In group 2, participants reported that they used water pipes 6.6 ± 1.8 times daily and the average duration of each water-pipe usage session was 29.7 ± 6.7 minutes. A family history of smoking was reported by 75.8%, 79.4% and 12.1% in groups 1, 2 and 3, respectively. Toothbrushing twice daily was reported by 84.8% in group 3 and 18.2% and 26.5% in groups 1 and 2, respectively. None of the individuals in groups 1 and 2 flossed their teeth and 12.1% (n=4) individuals in group 3 reported that they used dental floss once every day (Table 1).

**Table 1:** Demographics of the study population

Parameters	Group 1	Group 2	Group 3
Participants (n)	33	34	33
Gender (male)	25	30	30
Mean age in years	47.5 ± 10.5	45.4 ± 9.6	45.1 ± 9.2
Cigarette smoking (pack years)	9.4 ± 3.9	NA	NA
Duration of water-pipe usage	NA	13.5 ± 3.3 years	NA
Daily frequency of water-pipe usage (no. of times daily)	NA	6.6 ± 1.8 times daily	NA
Duration of each session of water-pipe usage (in minutes)	NA	29.7 ± 6.7 minutes	NA
Family history of smoking (%)	25 (75.8%)	27 (79.4%)	3 (9.1%)
Twice daily toothbrushing (%)	6 (18.2%)	9 (26.5%)	28 (84.8%)
Once daily flossing (%)	None	None	4 (12.1%)

Group 1: cigarette smokers; group 2: water-pipe users; group 3: non-smokers; NA: not applicable.

### Clinical and Radiographic Parameters

Statistically significantly elevated values were observed in PI (p<0.05), GI (p<0.05), PD (p<0.05), and mesial (p<0.05) and distal (p<0.05) MBL among patients in groups 1 and 2 when compared to individuals in group 3. The CAL scores for groups 1 and 2 were 3.45 ± 0.97 and 3.62 ± 1.2 mm, respectively, while none of the individuals in the control group exhibited CAL. The number of missing teeth was statistically significantly higher in groups 1 (p<0.05) and 2 (p<0.05) compared to group 3. No statistically significant differences were observed in PI, GI, PD, CAL, missing teeth, and mesial and distal MBL among patients in groups 1 and 2. Detailed results are provided in Table 2.

**Table 2:** Periodontal parameters

Parameters	Group 1	Group 2	Group 3
Plaque index	0.83 ± 0.22*	0.78 ± 0.18†	0.11 ± 0.04
Gingival index	0.32 ± 0.25	0.34 ± 0.27	0.2 ± 0.1
Probing depth	4.94 ± 0.95 mm*	4.42 ± 0.81 mm†	1.41 ± 0.7 mm
Clinical attachment loss	3.45 ± 0.97 mm	3.62 ± 1.2 mm	NA
Marginal bone loss (mesial)	4.26 ± 0.72 mm*	4.14 ± 1.1 mm†	0.9 ± 0.6 mm
Marginal bone loss (distal)	4.31 ± 0.5 mm*	4.21 ± 0.95 mm†	0.71 ± 0.4 mm
Missing teeth	13.5 ± 2.5 teeth*	11.2 ± 1.3 teeth†	2.8 ± 0.4 teeth

*Compared with group 3 (p<0.05); † Compared with group 3 (p<0.05).

### Unstimulated Whole Salivary Flow Rate and PgE2 Levels

There was no statistically significant difference in flow rates of saliva among individuals in groups 1, 2 and 3. The PgE2 levels were markedly higher among patients in groups 1 (p < 0.05) and 2 (p<0.05) in comparison to group 3. There was no statistically significant difference in PgE2 levels between participants in groups 1 and 2, as shown in Table 3.

**Table 3:** Periodontal parameters

Parameters	Group 1	Group 2	Group 3
Unstimulated whole salivary flow rate	0.34 ± 0.1 ml/min	0.32 ± 0.11 ml/min	0.33 ± 0.14 ml/min
Prostaglandin E2 levels	231.5 ± 66.3 pg/ml*	243.1 ± 69.3 pg/ml*	76.6 ± 10.6 pg/ml

*Compared with group 3 (p<0.05).

### Regression Analyses

**Fig 2:**
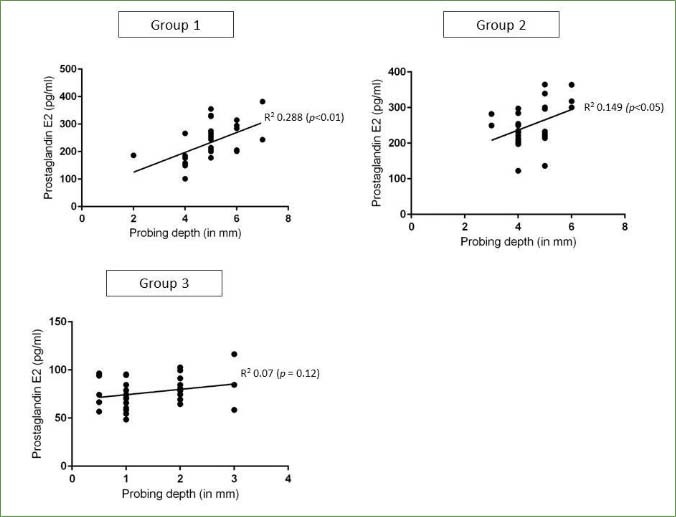
Correlation between pack-years and duration of water-pipe smoking and whole salivary PgE2 levels in groups 1 and 2.

**Fig 3:**
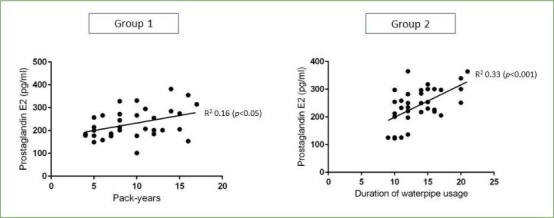
Correlation between PD and whole salivary PgE2 levels in the study groups.

In groups 1 and 2, there was a statistically significant correlation between pack-years, duration of water-pipe smoking and PD and whole salivary PgE2 levels, as shown in Figs 2 and 3. There was no statistically significant correlation between age, gender, family history of smoking, UWSFR, PI, GI, CAL, MBL, duration of each session of water-pipe usage and oral-hygiene maintenance protocols or whole salivary PgE2 levels in all groups.

## DISCUSSION

The findings of the present study are in line with a body of prior research that has consistently demonstrated a higher incidence of periodontal inflammatory conditions among CS when compared to non-smokers.^9,14^ Furthermore, the current investigation aligns with previous studies,^12,34^ emphasising that water-pipe usage cannot be regarded as a safe alternative to CS in the context of oral health. Specifically, our results support the notion that individuals who use water pipes are, to a similar extent, as likely as CS to exhibit periodontal diseases when compared with non-smokers. In a molecular cross-sectional epidemiological investigation led by Zhong et al,^44^ the authors explored the relationships between mean concentrations of PgE2 in the gingival crevicular fluid (GCF) and clinical indicators of periodontal disease in a community-based sample comprising 6277 adults aged 52 to 74 years. The results revealed a positive correlation between GCF PgE2 levels and periodontal PD and gingival bleeding in the studied population.^44^ However, a meticulous examination of indexed literature has shown that the comprehensive assessment of whole salivary PgE2 concerning its association with periodontitis and exposure to combustible nicotine products has not been explored. Consequently, the current study focused on investigating the expression of PgE2 in UWS and its correlation with demographic, clinicoradiographic, and periodontal inflammatory parameters. In this context, the outcomes reported by Zhong et al^44^ should be mentioned, that periodontal inflammatory parameters were more severe, and whole salivary PgE2 levels were statistically significantly higher in CA (group 1) and water-pipe users (group 2) compared to non-smokers (group 3). Additionally, in agreement with Zhong et al,^44^ the current results also establish a statistically significant correlation between whole salivary PgE2 levels, PD and duration of smoking and water-pipe usage in groups 1 and 2 compared to group 3. An experimental study conducted by Liu et al^30^ is also pertinent, as it explores the role of the PgE2 pathway in orchestrating tissue damage. The findings of that investigation^30^ showed that PgE2 pathways mediate oxidative stress (OS) in systemic epithelial cells. Furthermore, PgE2 is produced by various cell types, including inflammatory cells, such as macrophages and fibroblasts, in response to inflammatory stimuli, e.g., bacterial pathogens associated with periodontal infections.^2^ These findings imply that the increased expression of PgE2 in the UWS of both CS and water-pipe users may represent an adaptive response aimed at mitigating periodontal inflammation triggered by intraoral exposure to tobacco smoke. In this context, assessment of whole salivary PgE2 may serve as a potential biomarker of periodontal inflammation in susceptible patient populations.

A remarkable observation in the present study was that a statistically significant correlation was noted between whole salivary PgE2 levels and duration of water-pipe usage and CS. Moreover, a correlation between these groups and PD was also evident (Figs 2 and 3). This finding implies that the extent of exposure to water-pipe usage and CS may influence the salivary PgE2 levels, indicating a potential role of PgE2 in the oral response to these forms of tobacco use. Furthermore, the correlation between these groups and PD suggests a potential association between the duration of water-pipe usage, CS, and the severity of periodontal disease, as reflected by PD. This supports existing literature that establishes a connection between tobacco use and an increased risk of periodontal diseases. It is important to note that while the observed correlations provide valuable insights, further research is warranted to elucidate the underlying mechanisms and causative relationships. Additionally, considering the multifactorial nature of periodontal diseases, future studies should explore the interplay between PgE2, tobacco use, and other relevant factors to comprehensively understand their contributions to oral health outcomes.

All individuals in group 1 were “light” CS as they had a smoking history of approximately 9 pack-years. Moreover, subgingival microbiota were not investigated in the study groups. It has been reported that subgingival microbial counts such as red complex bacteria (RCB) are higher in individuals who habitually use combustible nicotinic product compared with non-smokers.^16,17^ It is therefore hypothesised that subgingival counts of pathogenic microbes including the RCB are higher in heavy CS compared with mild and moderate CS and non-smokers, and that whole salivary PgE2 levels are modulated by smoking pack-years and subgingival microbial colonisation in vulnerable patients. In this study, it must also be mentioned that patients with systemic diseases such as poorly-controlled DM were excluded. It is known that persistent hyperglycemia, a classical manifestation of poorly-controlled DM is associated with delayed healing.^28^ Moreover, in an experimental study on mouse models, Tang et al^42^ showed that a state of persistent hyperglycemia induces OS in periodontal tissues including the periodontal ligament, which augments alveolar bone loss.^42^ It is hypothesised that the periodontal clinicoradiographic status as well as outcomes of periodontal interventions such as surgical and non-surgical periodontal therapy are compromised in tobacco-smokers with poorly-controlled DM compared to systemically healthy, non-smoking individuals. Further studies are needed to test these hypotheses.

It is noteworthy that participants in group 3 exhibited a higher percentage of twice-daily toothbrushing compared to individuals in groups 1 and 2. Research indicates that lower educational attainment and disadvantaged living conditions serve as risk factors for suboptimal oral-hygiene maintenance and the development of periodontal diseases.^24^ We posit that advancements in lifestyle and education, particularly among users of tobacco products, may contribute to improvements in both their oral and systemic health status. The hypothesis suggests that targeted interventions aimed at enhancing lifestyle choices and educational opportunities, especially for individuals using tobacco products, could lead to positive outcomes in oral and overall health. Implementing routine community-based health education programs that emphasise the adverse effects of nicotine product usage, underscore the importance of oral hygiene maintenance, and advocate for regular dental visits may play a crucial role in elevating the overall health and oral health-related quality of life within the community.

## CONCLUSION

There is no difference in periodontal clinicoradiographic status and whole salivary PgE2 levels among CS and water-pipe users; however, these parameters are statistically significantly worse in CS and water-pipe users than in non-smokers.

## References

[ref1] Aguirre CG, Madrid J, Leventhal AM (2015). Tobacco withdrawal symptoms mediate motivation to reinstate smoking during abstinence. J Abnorm Psychol.

[ref2] Airila-Månsson S, Söder B, Kari K, Meurman JH (2006). Influence of combinations of bacteria on the levels of prostaglandin E2, interleukin-1beta, and granulocyte elastase in gingival crevicular fluid and on the severity of periodontal disease. J Periodontol.

[ref3] Akram Z, Al-Kheraif AA, Kellesarian SV, Vohra F, Javed F (2018). Comparison of oral Candida carriage in waterpipe smokers, cigarette smokers, and non-smokers. J Oral Sci.

[ref4] Ali D, Al-Yahya QM, Baskaradoss JK (2023). Peri-implant inflammation in waterpipe users and cigarette smokers: an observational study. Int Dent J.

[ref5] Ali D, AlAhmari F, Mikami T, Baskaradoss JK (2022). Increased expression of advanced glycation endproducts in the gingival crevicular fluid compromises periodontal status in cigarette-smokers and waterpipe users. BMC Oral Health.

[ref6] AlMubarak AM, Alqutub MN, Javed F, Vohra F, Abduljabbar T (2022). Whole salivary cotinine levels and interleukin 1-β levels among young adults involuntarily exposed to vapor from electronic nicotine delivery systems. Oral Health Prev Dent.

[ref7] Alqahtani F, Alqahtani M, Albaqawi AH, Al-Kheraif AA, Javed F (2019). Comparison of cotinine levels in the peri-implant sulcular fluid among cigarette and waterpipe smokers, electronic-cigarette users, and nonsmokers. Clin Implant Dent Relat Res.

[ref8] Ameringer KJ, Leventhal AM (2015). Psychological symptoms, smoking lapse behavior, and the mediating effects of nicotine withdrawal symptoms: A laboratory study. Psychol Addict Behav.

[ref9] Apatzidou DA (2000). The role of cigarette smoking in periodontal disease and treatment outcomes of dental implant therapy. Periodontol.

[ref10] Armitage GC (2000). Periodontal diagnoses and classification of periodontal diseases. Periodontol.

[ref11] Bergström J (2004). Tobacco smoking and chronic destructive periodontal disease. Odontology.

[ref12] Bibars AR, Obeidat SR, Khader Y, Mahasneh AM, Khabour OF (2015). The effect of waterpipe smoking on periodontal health. Oral Health Prev Dent.

[ref13] Caton JG, Armitage G, Berglundh T, Chapple ILC, Jepsen S, Kornman KS (2018). A new classification scheme for periodontal and peri-implant diseases and conditions - Introduction and key changes from the 1999 classification. J Clin Periodontol.

[ref14] Chaffee BW, Couch ET, Vora MV, Holliday RS (2000). Oral and periodontal implications of tobacco and nicotine products. Periodontol.

[ref15] Cheng H, Huang H, Guo Z, Chang Y, Li Z (2021). Role of prostaglandin E2 in tissue repair and regeneration. Theranostics.

[ref16] Feres M, Bernal M, Matarazzo F, Faveri M, Duarte PM, Figueiredo LC (2015). Subgingival bacterial recolonization after scaling and root planing in smokers with chronic periodontitis. Aust Dent J.

[ref17] Grossi SG, Goodson JM, Gunsolley JC, Otomo-Corgel J, Bland PS, Doherty F (2007). Mechanical therapy with adjunctive minocycline microspheres reduces red-complex bacteria in smokers. J Periodontol.

[ref18] Gümüş P, Nizam N, Nalbantsoy A, Özçaka Ö, Saliva Buduneli N (2017). Serum levels of interleukin-21, -33 and prostaglandin E2 in patients with generalised aggressive or chronic periodontitis. Oral Health Prev Dent.

[ref19] Gümüş P, Öztürk V, Bozkurt E, Emingil G (2016). Evaluation of the gingival inflammation in pregnancy and postpartum via 25-hydroxy-vitamin D3, prostaglandin E2 and TNF-α levels in saliva. Arch Oral Biol.

[ref20] Hajishengallis G, Chavakis T (2021). Local and systemic mechanisms linking periodontal disease and inflammatory comorbidities. Nat Rev Immunol.

[ref21] Javed F, Al-Askar M, Samaranayake LP, Al-Hezaimi K (2013). Periodontal disease in habitual cigarette smokers and nonsmokers with and without prediabetes. Am J Med Sci.

[ref22] Javed F, Al-Kheraif AA, Al Amri MD, Alshehri M, Vohra F, Al-Askar M (2015). Periodontal status and whole salivary cytokine profile among smokers and never-smokers with and without prediabetes. J Periodontol.

[ref23] Javed F, Al-Kheraif AA, Salazar-Lazo K, Yanez-Fontenla V, Aldosary KM, Alshehri M (2015). Periodontal inflammatory conditions among smokers and never-smokers with and without type 2 diabetes mellitus. J Periodontol.

[ref24] Javed F, Näsström K, Benchimol D, Altamash M, Klinge B, Engström PE (2007). Comparison of periodontal and socioeconomic status between subjects with type 2 diabetes mellitus and non-diabetic controls. J Periodontol.

[ref25] Javed F, AL SS, BinShabaib MS, Gajendra S, Romanos GE, Rahman I (2017). Toxicological impact of waterpipe smoking and flavorings in the oral cavity and respiratory system. Inhal Toxicol.

[ref26] Johnson TM (2017). Smoking and periodontal disease. US Army Med Dep J.

[ref27] Kawabata A (2011). Prostaglandin E2 and pain--an update. Biol Pharm Bull.

[ref28] Ko KI, Sculean A, Graves DT (2021). Diabetic wound healing in soft and hard oral tissues. Transl Res.

[ref29] Lee YH, Shin MH, Kweon SS, Choi JS, Rhee JA, Ahn HR (2011). Cumulative smoking exposure, duration of smoking cessation, and peripheral arterial disease in middle-aged and older Korean men. BMC Public Health.

[ref30] Liu Y, Zhou L, Lv C, Liu L, Miao S, Xu Y (2023). PGE2 pathway mediates oxidative stress-induced ferroptosis in renal tubular epithelial cells. Febs j.

[ref31] Loe H, Silness J (1963). Periodontal disease in pregnancy. I. Prevalence and severity. Acta Odontol Scand.

[ref32] Mokeem SA, Abduljabbar T, Al-Kheraif AA, Alasqah MN, Michelogiannakis D, Samaranayake LP (2019). Oral Candida carriage among cigarette- and waterpipe-smokers, and electronic cigarette users. Oral Dis.

[ref33] Mokeem SA, Alasqah MN, Michelogiannakis D, Al-Kheraif AA, Romanos GE, Javed F (2018). Clinical and radiographic periodontal status and whole salivary cotinine, IL-1β and IL-6 levels in cigarette- and waterpipe-smokers and E-cig users. Environ Toxicol Pharmacol.

[ref34] Natto S, Baljoon M, Dahlén G, Bergström J (2005). Tobacco smoking and periodontal microflora in a Saudi Arabian population. J Clin Periodontol.

[ref35] Papapanou PN, Sanz M, Buduneli N, Dietrich T, Feres M, Fine DH (2018). Periodontitis: Consensus report of workgroup 2 of the 2017 World Workshop on the Classification of Periodontal and Peri-Implant Diseases and Conditions. J Periodontol.

[ref36] Qasim H, Alarabi AB, Alzoubi KH, Karim ZA, Alshbool FZ, Khasawneh FT (2019). The effects of hookah/waterpipe smoking on general health and the cardiovascular system. Environ Health Prev Med.

[ref37] Ramôa CP, Eissenberg T, Sahingur SE (2017). Increasing popularity of waterpipe tobacco smoking and electronic cigarette use: Implications for oral healthcare. J Periodontal Res.

[ref38] Rodríguez M, Domingo E, Municio C, Alvarez Y, Hugo E, Fernández N (2014). Polarization of the innate immune response by prostaglandin E2: a puzzle of receptors and signals. Mol Pharmacol.

[ref39] Sakata D, Yao C, Narumiya S (2010). Prostaglandin E2, an immunoactivator. J Pharmacolog Sci.

[ref40] Sánchez GA, Miozza VA, Delgado A, Busch L (2013). Salivary IL-1β and PGE2 as biomarkers of periodontal status, before and after periodontal treatment. J Clin Periodontol.

[ref41] Silness J, Loe H (1964). Periodontal disease in pregnancy. II. Correlation between oral hygiene and periodontal condtion. Acta Odontol Scand.

[ref42] Tang L, Li T, Chang Y, Wang Z, Li Y, Wang F (2022). Diabetic oxidative stress-induced telomere damage aggravates periodontal bone loss in periodontitis. Biochem Biophys Res Commun.

[ref43] Wilson DJ, DuBois RN (2022). Role of Prostaglandin E2 in the Progression of Gastrointestinal Cancer. Cancer Prev Res.

[ref44] Zhong Y, Slade GD, Beck JD, Offenbacher S (2007). Gingival crevicular fluid interleukin-1beta, prostaglandin E2 and periodontal status in a community population. J Clin Periodontol.

